# Participatory Epidemiology: Principles, Practice, Utility, and Lessons Learnt

**DOI:** 10.3389/fvets.2020.532763

**Published:** 2020-11-04

**Authors:** Robyn G. Alders, Syed Noman Ali, Aluma Araba Ameri, Brigitte Bagnol, Tarni L. Cooper, Ahmad Gozali, M. M. Hidayat, Elpidius Rukambile, Johanna T. Wong, Andrew Catley

**Affiliations:** ^1^Kyeema Foundation, Brisbane, QLD, Australia; ^2^Kyeema Foundation, Maputo, Mozambique; ^3^Center for Universal Health, Chatham House, London, United Kingdom; ^4^Development Policy Center, Australian National University, Canberra, NSW, Australia; ^5^Food and Agriculture Organization of the United Nations Animal Health, Jakarta, Indonesia; ^6^Livestock Department, Government of Sindh, Karachi, Pakistan; ^7^Private Consultant, Juba, South Sudan; ^8^Department of Anthropology, University of the Witwatersrand, Johannesburg, South Africa; ^9^School of Veterinary Science, The University of Queensland, Brisbane, QLD, Australia; ^10^International Livestock Research Institute, Hanoi, Vietnam; ^11^Centre for Communication and Social Change, School of Communication and Arts, The University of Queensland, Brisbane, QLD, Australia; ^12^Directorate General of Livestock and Animal Health Services, Ministry of Agriculture, Jakarta, Indonesia; ^13^Tanzania Veterinary Laboratory Agency, Dar es Salaam, Tanzania; ^14^Faculty of Science, School of Life and Environmental Sciences, University of Sydney, Sydney, NSW, Australia; ^15^Friedman School of Nutrition Science and Policy at Tufts University, Feinstein International Center, Boston, MA, United States

**Keywords:** participatory disease surveillance, medical anthropology, emerging infectious disease, One Health, participatory impact assessment

## Abstract

Participatory epidemiology (PE) evolved as a branch of veterinary epidemiology and has been largely employed for the control and early warning of infectious diseases within resource-limited settings. It was originally based on combining practitioner communication skills with participatory methods to facilitate the involvement of animal caretakers and owners (embracing their knowledge, experience, and motivations) in the identification and assessment of animal disease problems, including in the design, implementation, monitoring and evaluation of disease control programs, policies, and strategies. With the importance of understanding social perceptions and drivers receiving increasing recognition by epidemiologists, PE tools are being adapted for an increasingly wide range of settings and endeavors. More recently, PE tools have been adapted for use in food and nutrition security programs, One Health activities, wildlife disease surveillance and as part of mixed-methods research across a range of socio-economic settings. This review describes the evolution of PE (in relation to veterinary epidemiology and briefly in relation to public health epidemiology), the underpinning philosophy and principles essential to its effective application and the importance of gender-sensitive approaches and data triangulation, including conventional confirmatory testing. The article also provides illustrative examples highlighting the diversity of approaches and applications of PE, hallmarks of successful PE initiatives and the lessons we can learn when these are missing. Finally, we look forward, describing the particular utility of PE for dealing with emerging infectious diseases, gaining attention of field-level cross-sector officials who can escalate concerns to a higher level and for continuing to raise the voices of those less-heard (such as women, minority groups, and remote communities with limited exposure to formal education) in defining the problems and planning activities that will likely impact directly on their well-being and livelihoods.

## Introduction

Participatory epidemiology (PE) evolved as a branch of veterinary epidemiology and has been largely employed for the control and early warning of infectious diseases within resource-limited settings (1–4). These approaches and methods are derivatives of participatory appraisal and are useful in several conditions where the conventional epidemiological approaches do not provide the adequate level of understanding of the existing situation important for designing appropriate intervention. It was originally based on combining practitioner communication skills with participatory methods to facilitate the involvement of animal caretakers and owners (embracing their knowledge, experience, and motivations) in the identification and assessment of animal disease problems, including the design, implementation, monitoring and evaluation of disease control programs, policies, and strategies. This review describes the evolution of PE (in relation to veterinary epidemiology and briefly in relation to public health epidemiology), the underpinning philosophy and principles essential to its effective application, and highlights the importance of gender-sensitive approaches and data triangulation, including conventional confirmatory testing. It discusses the importance of understanding social perceptions and drivers, which is receiving increasing recognition by epidemiologists, and provides examples as to how PE tools are being adapted for an increasingly wide range of settings and endeavors, including: use in food and nutrition security programs (5–7); One Health activities (8); wildlife disease surveillance (9); gender analysis (10, 11); communication (12, 13); and for monitoring and evaluation (14).

## History and Definition Evolution

Paulo Freire (15) in “Pedagogy of the oppressed” advocated for a dialogue, and a participatory process for social transformation. By the late 1980s, there was a shift toward a more participatory approach to research, communication and extension services, particularly in the context of development activities. Consequently, participatory methodologies have been increasingly used in agricultural and livestock research development programs. Their use emerged in response to the failure of “normal” science to yield sustainable improvements to production and livelihoods in resource-limited, rural settings because of its inability to describe and intervene effectively in the complex and changing experiences of farmers and others involved in rural development (16). Early approaches were centralized and top-down (17). This top-down approach was unidirectional; initiated by the educated, expert, or intellectual (the “haves”), and directed toward the uneducated or ignorant (the “have nots”). This approach aimed to educate, convince or persuade individuals that their practices were wrong, and they should implement “modern” techniques. Chambers (18), publicized the idea of “putting the last first” and development organizations and extension services started to adopt some of these concepts. This led to demand-led extension, a process by which the information, advice and other extension services should be tailored to the expressed demands of the clients or users of the service (19–21). In participatory studies, knowledge is considered subjective and is generated through practical understanding of community practices (22). Subjective quality criteria are measured by the extent of individual's practical experience which leads to human improvement, hence the values of both the researcher and the participant are automatically brought into the research process.

Analysis of prior usage of participatory methodologies by Pretty (16) revealed at least seven different types of participation (Table 1) and lead to the recommendation that the term “participation” should always come with the appropriate qualification. Our detailed review of PE was compiled with these different levels of participation in mind.

**Table 1:** A typology of participation: how people participate in development programs and projects (16).

Passive participation	People participate by being told what is going to happen or has already happened. It is a unilateral announcement by an administration or project management without any listening to people's responses.
Participation in information giving	The information being shared belongs only to external professionals. People participate by answering questions posed by extractive researchers using questionnaire surveys or such similar approaches. People do not have the opportunity to influence proceedings, as the findings of the research are neither shared nor checked for accuracy.
Participation by consultation	People participate by being consulted, and external agents listen to views. These external agents define both problems and solutions and may modify these in the light of people's responses. Such a consultative process does not concede any share in decision making, and professionals are under no obligation to take on board people's views.
Participation for material benefits	People participate by providing resources such as labor, in return for food, cash or other material incentives. Much on-farm research falls in this category, as farmers provide the fields but are not involved in experimentation or the process of learning. It is very common to see this called participation, yet people have no stake in prolonging activities when incentives end.
Functional participation	People participate by forming groups to meet pre-determined objectives related to the project, which can involve the development or promotion of externally initiated social organization. Such involvement tends not to be at early stages of project cycles or planning, but rather after major decisions have already been made. These institutions tend to be dependent on external initiators and facilitators but may become self-dependent.
Interactive participation	People participate in joint analysis, which leads to action plans and the formation of new local institutions or the strengthening of existing ones. It tends to involve interdisciplinary methodologies that seek multiple objectives and make use of systematic and structured learning processes. These groups take control/ownership over local decisions, and so people have a stake in maintaining structures or practices.
Self-mobilization	People participate by taking initiatives independent of external institutions to change systems. Such self-initiated mobilization and collective action may or may not challenge existing inequitable distributions of wealth and power.

In 2000, Mariner and Paskin defined PE as “an emerging field that is based on the use of participatory techniques for the harvesting of qualitative epidemiological intelligence contained within community observations, existing veterinary knowledge and traditional oral history.” Subsequently, Catley et al. (2) proposed a refined working definition of PE, i.e., “the systematic use of participatory approaches and methods to improve understanding of diseases and options for animal disease control.” This working definition referred to “both a ‘participatory approach' and ‘participatory methods,' indicating that an understanding of both approach and methods are needed to define PE.” Catley et al. (2) further proposed that “the term ‘participatory' in PE is used to refer to the essential involvement of communities in defining and prioritizing veterinary-related problems, and in the development of solutions to service delivery, disease control, or surveillance…. use of the term PE that does not involve communities in these ways is considered to be a misnomer.” In 2017, as part of a study of the major applications of PE in animal health, a modification of the Catley et al. (2) definition was proposed: “Participatory epidemiology is the systematic use of approaches and methods that facilitate the empowerment of people to identify and solve their health needs. It should promote the participation of people, leading to a shared learning environment that improves the understanding of their risk perception, health risks and options for surveillance, control, and health evaluation in populations. It should be conducted by professionals on equal partnership among all involved in the activity and with mutual respect and trust, ensuring acceptability and a sense of ownership” (3). This same study highlighted the utility of PE techniques in developing informed animal health policies by facilitating dialogue between communities and animal health officials in relation to disease prioritization. A 2020 review of PE disease control activities in pastoralist areas of Africa (4) examined the Allepuz et al. (3) modified definition by exploring the concept of empowerment within communities with significant socio-economic differentiation. Marked differences in wealth between households (4) and within households (23) have a significant effect on disease impacts and priorities and prevention and control preferences. Ensuring that PE techniques are applied through a gender-sensitive lens is crucial to achieving just and sustainable actions (2, 23).

The element of responsiveness or action combined with community engagement appears to set the PE employed within animal health apart from PE as employed within the public health arena. For example, “participatory” epidemiology has been used to refer to autonomous surveillance of social media for potential disease events (24). Bach et al. (25) conducted a review of the contribution of participatory research to epidemiology, emphasizing how participatory approaches can enhance common epidemiological approaches. The importance of the dissemination of findings was stressed by Bach et al. (25) but the need to actively work with communities to develop solutions appeared to be lacking in the review.

## Approaches, Methodologies, and Tools

Rapid rural appraisal (RRA) was a commonly employed, early approach to conducting a discrete study in one or more rural communities. These RRA studies were typically conducted within a week by a multidisciplinary team of researchers looking at a set of issues that were clearly defined by the study objectives (26). Participatory rural appraisal (PRA) subsequently emerged as an extended process that involves the collection of information and its eventual use by the community as it plans further activities (27). The aim of PRA is to stimulate a learning process and knowledge generation based on community members' experience to define priorities, and collect, analyse and interpret data (28–30). Participants are seen as the owners of the methods and outcomes of the appraisal. Participatory action research (PAR) goes a step further by utilizing the knowledge and understanding of community members as a point of reference to generate a participatory learning framework and actions. Research participants bridge the gap between the researcher and the researched by engaging in the data collection and scrutiny, and determination of the achievement trend of the research (22, 31). The ultimate goal of PAR is practical knowledge generation, making sure that the knowledge is made available and used for the transformation and empowerment of the individual participants and community at large (32). Participatory studies, including those beyond PE, deploy a wide range of techniques for data collection including but not limited to personal interviews, focus group discussions, observation, free listing, ranking, pair-wise ranking, causal flow analysis, open-ended stories, genograms, role playing, body mapping, and photo voice (30, 33, 34). The tools used for data collection in PRA and PAR should ensure gender inclusion and reduced gaps between the literate and illiterate to increase the chances of achieving equal access during information generation and sharing.

Table 2 summarizes the range of PE methodologies and tools that are now regularly in use in the field across a range of settings. Key to the successful use of these methods is an understanding of and commitment to: (i) the principles of adult learning (i.e., adult learners have different experiences, perceptions, problems and needs, and activities are more effective if trainers and PE practitioners understand how and why adults learn), (ii) triangulation (i.e., using more than one method to collect data on the same topic to verify findings, including multiple qualitative sources and participants, the use of secondary documentation, clinical examination, and laboratory diagnostic tests), and (iii) laboratory diagnostic support (i.e., in cases of livestock disease investigation, the use of PE tools needs to be accompanied by laboratory confirmation as it is not enough to rely on data collection using PE tools only).

**Table 2:** An overview of the most commonly used PE methods and tools used to obtain specific information.

**Method**	**Tools**	**Examples of data gathered**
Informal interviewing (semi-structured)	Key informant interviews Focus-group discussions	Personal and group accounts of disease history and impacts Identification of important stakeholders
Ranking and scoring	Simple ranking Pair-wise ranking Proportional piling Matrix scoring Wealth ranking	Preferred types of livestock reared Relative livestock ownership Relative importance of livestock to livelihoods
Visualization	Participatory mapping Venn diagrams Seasonal calendars Timelines	Ecosystem boundaries and natural resources Veterinary services Seasonal variations in livestock disease Infrastructure Timeline of disease emergence and associated events
Direct observation	Transect walks Walking surveys	Infrastructure available Local environment Local living and working conditions Potential drivers of disease (such as water bodies, animal movements and interactions) Distance examination of animals for signs of disease
Participatory disease surveillance	The entire suite of participatory tools listed above applied to the disease of interest (usually based on syndromic diagnosis)	Information to develop a case definition Existence of or estimate of prevalence, incidence, morbidity and/or mortality of disease of interest

A number of PE training documents are freely available online as are explanations of novel uses of these tools to tackle a range of animal, human, and One Health issues (Table 3).

**Table 3:** A compilation of PE methodology and training resources available free of charge online.

**PE component**	**Potential applications**	**Source**
Manual on participatory epidemiology	Action-oriented epidemiological intelligence collection and joint analysis	(35)
Participatory methodologies for use in pastoral areas	Disease surveillance in areas where animal healthcare and disease reporting systems are limited. To support the joint preparation of feasible and acceptable disease control strategies.	(1)
Participatory Epidemiology: a guide for trainers	Animal health surveys Problem analysis Disease detection Changing disease patterns Research	(36)
Participatory impact assessment	Participatory development of impact indicators by a range of stakeholders.	(37)
Participatory methodologies for family poultry production through a gender lens	To improve husbandry and biosecurity measures, and therefore health and production within small-scale chicken production systems for men and women farmers.	(38)
Trainer toolkit	A toolkit to assist in the implementation of introductory training programs in PE for adult students, including mid-career professionals.	(39)

## Implementation Experiences and Lessons Learnt From the Field

The selection of case studies below provide an insight into the utilization of PE approaches within countries, initially in relation to animal disease prevention, then in relation to the linkages between animal disease and human food and nutrition security and finally, antimicrobial resistance. While far from an exhaustive list, the studies were selected to provide a diverse overview of geographical, cultural, disease, and methodological applications of PE over the last 20 years.

The first case study from Pakistan provides an overview of how the application of PE evolved over time and demonstrates how participatory epidemiology helped to shift the focus from the three diseases targeted by international agencies, i.e., rinderpest, foot and mouth disease (FMD) and *peste des petits ruminants* (PPR) to haemorrhagic septicaemia which was of greater concern to local farmers. The second case study from Sudan illustrates the variety of uses of PE and how it contributes to strengthen under-resourced health services. The Indonesian case study highlights the evolution of PE methods from an animal health focus to a broader One Health framework. Moving on to more recent project-specific examples with greater integration of One Health, the fourth case study from Tanzania connects participatory animal health to participatory nutritional security and food safety through a gender lens. In Timor-Leste, gender-sensitive participatory approaches were used to learn about animal disease, household food choices and food safety, while the case study from Uganda revealed how underlying causes of malnutrition were related to gender issues. Finally, the last case study from Vietnam provides insight into the use of participatory tools improve our understanding of and response to antimicrobial resistance (AMR).

Most of the case studies reflect the experiences of country nationals employing PE in support of national priorities. The case studies also emphasize the importance of employing PE techniques as part of a suite of activities that address the limitations of PE while also indicating how PE can contribute to multi-sectoral and interdisciplinary studies of complex systems.

### Pakistan: From Global to Local Priorities

The Islamic Republic of Pakistan, situated in South Asia, is the world's fifth-most populous country with a population exceeding 212.2 million (40). The geography and climate of Pakistan are extremely diverse; it is divided into three major geographic areas: the northern highlands, the Indus River plain, and the Baluchistan Plateau. Correspondingly, the climate varies from tropical to temperate, with arid conditions in the coastal south. Rainfall varies greatly from year to year, and patterns of alternate flooding and drought are common in the plains of Pakistan. Arable agriculture is mainly confined to the central fertile plain of the Indus River. Livestock production is a noteworthy section of agriculture primarily active in the arid and hyper-arid zones with restricted resources. Three systems of production systems are reported nationally according to the agroecological zone, i.e., nomadic, transhumant and stationary, or family business (41–43). Veterinary services in the remote areas of the country are poor and livestock owners mostly depend upon local herbal treatment practiced by families for decades.

During the second half of the twentieth Century, countless rural poverty alleviation programs that were developed and executed in the country, mainly in remote areas, unfortunately failed because of the gap between the farmers' views about their requirements and the understanding of the agencies that developed the programs (44). In the livestock sector, poor disease awareness, and reporting systems contributed to gaps in the design and implementation of animal disease control and eradication strategies as highlighted during the Global Rinderpest Eradication Program (45). The success stories of the participatory disease surveillance (PDS) active surveillance method employed in Africa (described in more detail in the case study on South Sudan below) prompted the Ministry of Food, Agriculture and Livestock, and Provincial Livestock Departments to introduce it into the country in support of transboundary animal disease (TAD) control. Participatory disease surveillance was implemented as a consultative process that proved to be valuable during the rinderpest eradication campaign from 1999 to 2007. Data obtained from PE was used to revise and improve rinderpest control methods and norms, both nationally and internationally (2, 46, 47). The PDS program greatly boosted the sensitivity of active clinical rinderpest surveillance and was pivotal to Pakistan's decision to declare provisional freedom from rinderpest to the OIE in January of 2003 (48). The integration of PDS with passive surveillance systems, based on reports from government and private veterinarians, enhanced their effectiveness by aggregating the number of cases detected for disease investigation and the timeliness of detection (47).

The occurrence of various important livestock diseases, particularly TADs such as Food and Mouth Disease (FMD; cattle and buffaloes) and *Peste des petits ruminants* (PPR) (sheep and goats) in the country were determined by applying different PDS tools. A full review of the data collected revealed that although FMD was the most prevalent disease, haemorrhagic septicaemia was considered the most important by farmers. Disease intelligence was gathered through various PE tools including visualization, scoring, and interview techniques (44, 46). Additional livestock health constraints documented during the field disease search program were mastitis, respiratory syndrome, intestinal parasite infestation, and buffalo pox. Gathering disease information through the application of participatory tools was a new approach in Pakistan. Initially, the majority of dairy farmers were hesitant about sharing their information and reluctant to actively participate in group discussions. Fortunately, as they came to understand that their indigenous knowledge was important and valued, it became relatively straight forward to obtain information pertinent to particular areas. The breadth and quality of data accrued through the application of PDS methodology has been valued by all livestock departments across the country. The estimation of disease prevalence and prioritization of their importance through PDS activities has helped to better plan and execute measures for the control/eradication of livestock diseases in different parts of the country. This approach was also found to be a practical option for obtaining reliable data that could be utilized by policy makers in their formulation of animal disease control and eradication in Pakistan (46). The most recent study was carried out in Tharparkar District of Sindh Province (44) in association with preventive vaccination against PPR disease.

Key lessons learnt to date in Pakistan, especially in relation to social behavior, include:

- Using a variety of exercises during interviews—such as scoring, mapping, and visualization—made it easier for farmers to share their point of view on various issues regarding livestock disease and the associated impact on their livelihoods;
- Some farmers hesitate to share information about infectious diseases in the presence of government veterinary staff;
- The PE approach was quite helpful when evaluating the disease situation in specific villages/areas. The interest of farmers/participants was very much evident during mapping, seasonal calendar and proportional piling exercises;
- Working with physical items that can be used to allocate preferences (e.g., stones/beans/seeds) during exercises was very effective in large groups and with key informants in rural areas. However, in peri-urban areas, livestock farmers preferred working with markers and charts;
- Through PDS activities, FMD, and PPR were found to be endemic throughout country. Farmers had been confusing PPR with contagious caprine pleuropneumonia and enterotoxaemia. Participatory disease surveillance teams confirmed PPR virus circulation serologically in villages of the country (46, 49);
- Foot and Mouth Disease and PPR were revealed, through participatory activities associated with the Rinderpest Eradication Program, to be causing socio-economic impacts that contributed to household poverty, malnutrition, starvation, and human health complications in rural areas of the country where mixed farming was common;
- Including female veterinarians in the PDS team was very successful in obtaining firsthand information from women who were directly involved in livestock management. Due to social restrictions male staff could not speak directly with women farmers; and
- Through the application of PDS tools (especially scoring and ranking tools), government veterinary services learnt that haemorrhagic septicaemia was of greater concern to farmers than the three diseases targeted by international agencies, i.e., rinderpest, FMD, and PPR.

### South Sudan: Community Engagement Strengthens Effectiveness of Under-Resourced Health Services

The Republic of South Sudan, one of the world's newest countries, covers an area roughly the same as France, and is bordered by Sudan, Central African Republic, Democratic Republic of Congo, Uganda, Kenya and Ethiopia. It has a variety of ecological zones, ranging from the flat savannah and flood plains around the Nile, and its tributaries, to the stony semi-arid region of the southeast to the rain forest of the undulating ironstone plateau of the west and south west. The climate fluctuates from very hot and dry in the dry season to hot and humid in the long rainy season when the low-lying areas are flooded, and every few years there are climatic extremes causing severe drought or floods (50).

In South Sudan, where population density is relatively low, infrastructure poor and ready access to human, and animal health services extremely limited in much of the country, a mix of consultative and interactive PE activities have played a vital role in disease control activities implemented through a One Health lens. “One Health” is the integrative effort of multiple disciplines working locally, nationally, and globally to attain optimal health for people, animals, and the environment (51). In the One Health space, consultative PE methodologies have been employed to conduct community-based surveillance and response systems for highly pathogenic avian influenza (HPAI) H5N1 from 2007 to 2009 [supported by USAID; (52)], and anthrax disease outbreak surveillance and control in humans and livestock in 2018 [including in South Sudanese refugees in Uganda; (53)]. The first wave of HPAI H5N1 outbreaks reported in poultry in Africa occurred in 2006, affecting eight African countries (Burkina Faso, Cameroon, Côte d'Ivoire, Djibouti, Egypt, Niger, Nigeria, and Sudan) in 2006 and three countries (Benin, Ghana, and Togo) in 2007 (54). A One Health approach was also adopted in South Sudan, involving veterinarians and human doctors, to conduct joint disease surveillance to investigate Rift Valley Fever (RVF) in the Lakes State. The approach resulted in the successful containment of RVF in livestock and human populations in the aforementioned state (55). Resources used to conduct interactive PE activities resulted not only in improved understanding of disease situations, they also simultaneously contributed to the development of collaborative approaches to disease surveillance and control.

Animal health studies utilizing PE to date have included applied research on a chronic wasting disease in cattle (called *liei* locally*)*, impact assessment of community-based animal health projects, and the application of participatory disease searching during the rinderpest eradication program (36). Participatory epidemiology was a crucial component of rinderpest disease searching in 2002–2007, and also for FMD (56) in remote areas where classical veterinary surveillance activities would have been difficult to implement. In each case, the methods used to obtain information from stakeholders (including livestock owners, livestock traders, local authorities in government offices, veterinarians, Community Animal Health Workers, youth, women, and men) depended on the objective of the disease control activity. For example, for rinderpest disease eradication, consultative participatory disease search methodologies were used to locate rinderpest virus foci in villages where veterinary services were limited during the civil war (1983–2005) (50). Professionals in South Sudan have applied a wide range of PE tools, including semi-structured interviews, seasonal calendars, simple ranking, proportional piling (PP), PP for morbidity and mortality, timelines, and participatory disease searching. It has been noted that the practical value of PE in South Sudan demonstrates that it should be valued as an essential skill for field veterinarians and livestock officers, working for government or NGOs (57).

Examples and utility of PE tools that are frequently employed in South Sudan include:

- Participatory Mapping (PM) is used when consulting livestock herders regarding seasonal grazing patterns and this information helps in designing vaccination campaigns with livestock owners in a participatory manner. Participatory mapping is mostly done at the beginning of focus group discussions (FGDs) and key informant interviews (KIIs) as way to break the ice and allow free interaction with the participants. It is especially useful when PE team members are visiting for the first time and know little about the area and the community leaders. Mapping provides key information concerning resources available (water, rivers, hills, and pastures), distance covered searching for grazing land, identifying neighboring community, villages, infrastructure like market points, social centers, and proximity of government and private services to the livestock owners. It can reveal the livestock species in the grazing sites, wildlife species, insecure areas where livestock theft is common, and conflict amongst neighbors, for example due to scarcity of water and pasture. All of this valuable information can be obtained in 1 h and helps to break down barriers between visiting teams and key informants, local leaders, local authority, and community members;
- Simple Ranking (SR) and PP are easy to use with individual participants, KIIs and FGDs. They provide good information for planning and further research. A SR exercise uses objects or cards that can be easily placed in order of priority based on information provided by participants. A PP exercise is conducted using cards or objects to represent issues with participants placing counters on issues proportionally to the size of the problem represented. The bigger the pile against particular card or object, the larger the concerns of participants regarding that problem. Simply ranking and PP exercises were done separately for each gender (i.e., men and women). It was found that when combining men and women into one discussion group, men tended to dominate and push their opinions above those raised by women, impeding the process of building consensus concerning key information discussed during the PE activities. Separating groups by gender can facilitate an environment where women can comfortably share their opinions and ideas.

These three PE tools (i.e., PM, SR, and PP) facilitated consultation and interaction with participants and generated considerable amounts of information, with elaborate details frequently emerging that the PE team used to probe further to generate useful data for disease outbreak investigation or project design and implementation. For example, in August 2006 in Juba, a PE team, composed of mainly veterinary officers, used participatory mapping to identify where poultry were dying with simple ranking used to gauge disease morbidity and mortality rates. These exercises were done with poultry owners who had reported sick chickens in Hai Jalaba. The sickness was perceived by livestock owners to be like Newcastle disease (ND). On the basis of the information provided by the owners the PE team suspected high pathogenic avian influenza (HPAI). As part of the triangulation process, samples were collected and dispatched to an OIE reference laboratory in the UK. Laboratory results confirmed the presence of HPAI H5N1 triggering the implementation of control activities.

### Indonesia: From Participatory Animal Health to One Health

The Republic of Indonesia is a country in Southeast Asia and Oceania consisting of more than 17,000 islands (58). It is the world's largest island country and the 14th largest country by land area. With over 267 million people, it is the world's fourth most populous country as well as the most populous Muslim-majority country. Java, the world's most populous island, is home to more than half of the country's population. Indonesia's size, tropical climate, and archipelagic geography support one of the world's highest levels of biodiversity (59).

In 2005, Indonesia became one of the Asian epicenters for human and animal HPAI H5N1 infections during the global pandemic (60). The Indonesian Ministry of Agriculture (MoA) and Ministry of Health (MoH), together with the Coordinating Ministry of Human Development and Cultural Affairs (MoHDCA) worked with the Food and Agriculture Organization of the United Nations (FAO) to control the H5N1 outbreak and continue to work on a pilot research and development program to identify sustainable strategies for strengthening capacities for One Health-focused, effective and sustainable prevention and control of targeted zoonoses and emerging infectious diseases (EIDs).

The participatory disease surveillance and response (PDSR) program, developed to tackle HPAI H5N1 in Indonesia, was an evolution of the consultative PDS system employed during the rinderpest eradication program in African countries and Pakistan (60). The first stage of the PDSR project commenced in January 2006 and focused on the detection and control of HPAI (H5N1) by separate PDS and participatory disease response (PDR) teams, primarily in extensively raised poultry kept by households within village settings. Lessons learned during the first phase were used to strengthen disease management during the second phase of the project (with field implementation starting in May 2008) by adapting technical approaches to HPAI disease control, increasing functional participation of key stakeholders, including relevant district, provincial and central government agencies, and focusing on the community level. The PDSR project concluded in September 2015 with the end of the FAO ECTAD Avian Influenza prevention and control program in Indonesia. The PDSR program focused almost entirely on HPAI with little to no attention paid to other diseases of poultry (61) as external donor funding was largely driven by the public health desire to prevent an avian influenza pandemic in humans.

Subsequently, the Indonesian Ministry of Environment and Forestry joined with the MoA, MoH, and MoHDCA in collaboration with the FAO to develop sustainable strategies for strengthening One Health-focused, effective and sustainable prevention and control of targeted endemic zoonoses (i.e., anthrax, avian influenza, and rabies) and EIDs (62). Commencing in 2017, an agreement between four collaborating ministries was signed and four pilot districts were selected covering different agro-ecological zones. Master trainers across human, animal, and wildlife health were nominated by their district agencies and together with a central training team, PE tools such as PM and PDS were adapted to support One Health field investigations of reports of zoonotic disease. The PE tools proved readily adaptable for use by wildlife health officers, many of whom did not have a background in veterinary science. Notable success was achieved in relation to the prevention and control of rabies, which is endemic in many provinces and is the most commonly reported zoonotic disease. Prior to One Health PE training, 99% (1152/1155) of bite cases were reported via the human health system only. After 18 months, 50% (431/855) of reported cases were managed via a One Health integrated bite case management protocol. Integrated bite case management reports (*n* = 431) increased from 1% before training to 50% post-One Health PE training (8). Overall, the Zoonoses Prevention and Control programme in Indonesia effectively incorporates the One Health approach within its multisectoral field operations and associated multisectoral communication and information sharing platforms. This programme provides a template for the operationalization of participatory One Health approaches in Indonesia and beyond. Moreover, through the involvement of economists in the One Health team, the programme was able to demonstrate that it was highly-cost effective, generating 6.6-14.4 USD in benefits per dollar invested (63). These findings together with effective intersectoral collaboration and positive feedback from communities lay the foundation for the development of the National Master Plan for the eradication of rabies using a One Health framework (64).

A One Health PE approach integrating human, animal, and wildlife health provides an opportunity to detect novel pathogens prior to their transmission to humans. The focus on existing zoonotic disease accommodated the immediate priorities of communities and frontline officers while simultaneously building effective disease prevention and control systems (8).

### Tanzania: From Participatory Animal Health to Participatory Nutritional Security and Food Safety

The United Republic of Tanzania is located in the eastern part of Africa and is about one tenth the size of the USA. Tanzania is bordered by Kenya and Uganda in the north; Burundi, Rwanda in the northwest; Democratic Republic of the Congo in the west; Malawi, and Zambia in the southwest; Mozambique in the south and Indian Ocean. Among the members of East Africa community, Tanzania has the largest population estimated at 58,552,845 and the lowest population density with almost one third of the population living in urban areas (65). From 1991 to 2015, the country has achieved significant decreases in stunting in children under 5 years of age from 50 to 35% and 22 to 12% of severe stunting in the same period (66). The Tanzania Demographic and Health Survey 2015–16 dataset indicates a prevalence of diarrhea in children under five of 12% (67), while diarhoea-specific mortality in the same age declined by 89% between 1980 and 2015 (68). Despite decreases in stunting and diarrhea-specific mortality, undernutrition and diarrhea in under five children are still important health problems and several efforts have been made through different platforms to overcome the problem including promoting animal and crop production and development of community-based educational packages.

An interdisciplinary and multi-sectoral team worked with local communities between 2014 and 2019 to strengthen traditional integrated livestock-crop systems in a semi-arid area of Central Tanzania with support from the Australian Center for International Agricultural Research (13, 69, 70). Representatives of the agriculture and health departments in Manyoni and Mpwapwa Districts were the key focal points of the project and were involved fully in the selection of participating wards. The village leaders from all three Wards were involved in the selection of men and women key informant interview (KII) and focus group discussion (FGD) participants and local project community workers. The community workers included village chicken vaccinators tasked with vaccinating household chickens during vaccination campaigns on a fee-for-service basis, the community assistants tasked with collecting fortnightly household data and enumerators who administered the questionnaire. Interventions targeted reduced mortality in extensively raised indigenous chickens through regular vaccination against Newcastle disease (ND) and constraints to the production and storage of nutrient-rich vegetables, grains, and pulses. Rural communities reliant on rain-fed crops often experience severe hunger periods immediately before the major harvesting season, when the previous year's stored grains have been exhausted or lost as a result of poor storage. Data was collected on human health and nutrition and household characteristics on a 6-monthly basis, livestock ownership on an annual basis, and chicken numbers and reports of diarrhea in children fortnightly as part of a cluster-randomized controlled trial involving children <24 months of age at the time of enrolment.

**Figure 1:**
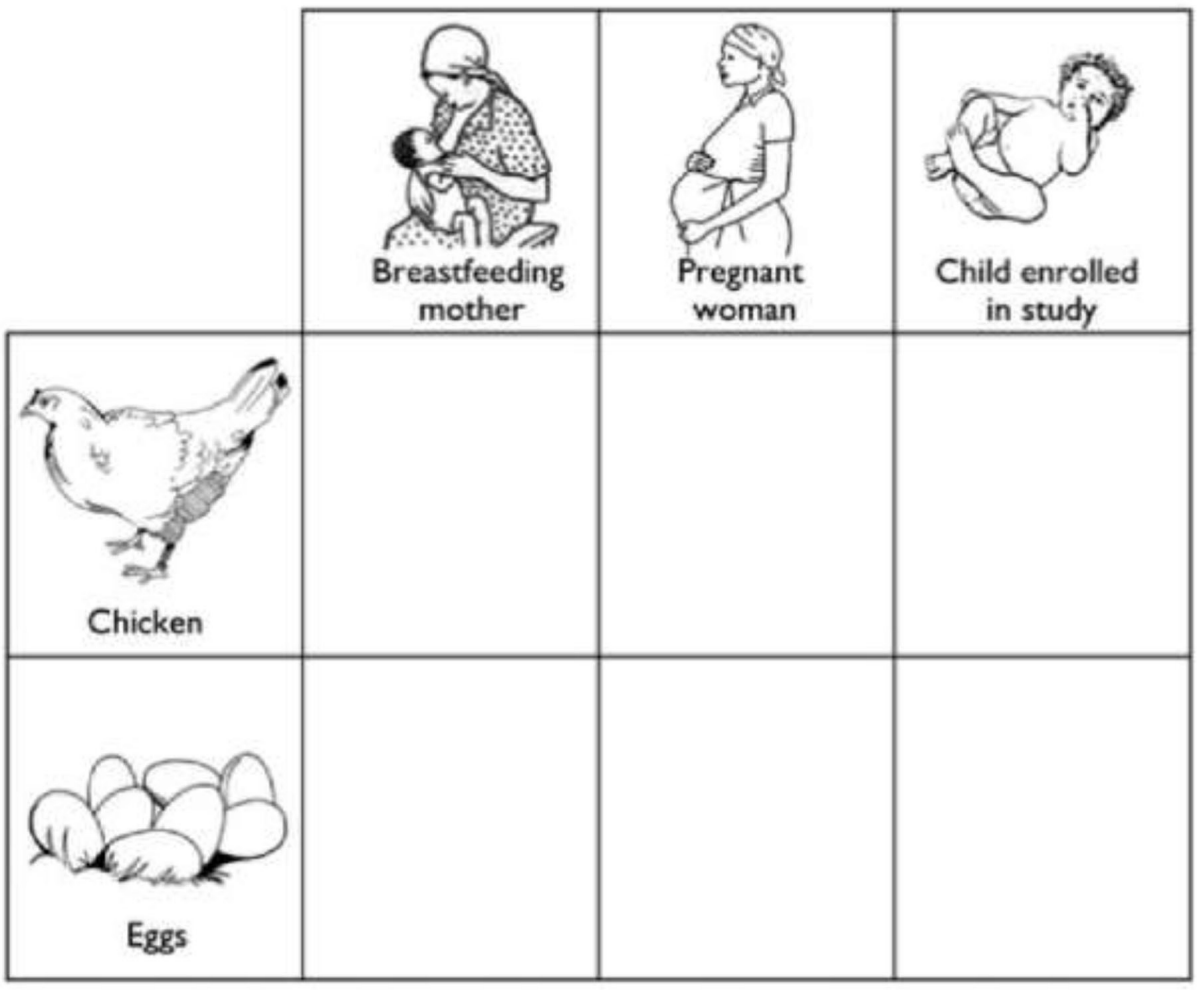
A copy of the pictorial record chart (with English translations of Swahili text) for completion by a household representative, to record the consumption of poultry products by children enrolled in the study, and any pregnant or breastfeeding women within the same household [Source: (5)].

To facilitate the active engagement of all enrolled households, pictorial record charts were distributed at 4 monthly intervals, in the months of August and December in 2014 and April, August, and December in 2015. The aim was to document the consumption of poultry products over a period of 4 consecutive weeks (5). This research tool was developed by anthropologist B. Bagnol for use in communities with low levels of literacy. It was adapted from an approach used in reproductive health research in Tanzania and Uganda (71, 72) to enable the involvement of those without an understanding of written language. Black and white line drawings depicting a chicken, eggs, an infant, a pregnant woman, and a breastfeeding mother were presented in a table layout (Figure 1). In advance of each data collection period, the locally-selected Community Assistants were trained to instruct a household representative to use a mark to record any meal containing chicken or egg consumed by the enrolled child or by a pregnant or breastfeeding woman in their household if present. Each household was visited by a Community Assistants visited on a weekly basis to review the pictorial charts and assist participants in recording any incomplete data as required. Triangulation of data was achieved using data from the visual diaries, annual gender disaggregated focus group discussions (11, 13) and quantitative survey tools.

Food safety is increasingly being recognized as a key component of food and nutrition security (73). Epidemiological studies indicate a significant association between unhygienic food handling and occurrence of childhood diarrhea diseases which suggests food contamination can result in acute and/or chronic gastrointestinal infections (74, 75). diarhoeal diseases, which in most cases occurs as a result of consumption of contaminated food, are associated with high morbidity and mortality, especially in children <5 years of age in many low- and middle-income countries (LMICs) (76). Environmental microbes are important sources of food contamination and the routes in which these microbes enter the human food chain vary from one setting to another. Poor water supply, sanitation services and unhygienic practices accompanied by extensive animal keeping which favor human-animal proximity increases the risks of environmental- and animal-associated microbes to enter the human food chain. A qualitative study with 10 KIIs and 8 gender-segregated FDGs (four FGDs with women and four with men) with an average of 8 participants was conducted in resource-poor settings in central rural Tanzania to explore challenges associated with water supply, sanitation services, hygiene practices, and animal husbandry, seen to be important underlying factors related to childhood diarrhea (77, 78). Also, community knowledge and perceptions of the causes and occurrence of diarrhea in children was examined as understanding this is essential to designing effective prevention and control of childhood gastrointestinal infections.

While the overarching 5-year study sought to achieve interactive participation, the food safety study employed participation by consultation listening to the views of the study participants on the components being studied. The researcher defined the problem and guided the participants through the discussion by ensuring equal opportunity for all participants until contributions had been exhausted. The questionnaire survey revealed that households switch water sources between the dry and rainy seasons, especially in areas with public taps. The survey findings alone were not enough to explain the reason for this shift. By engaging with FGD participants, it became clear that a large proportion of the households use ground water (rivers, pond, and streams) during the rainy season because it is free and convenient; whereas during the dry season most households use public taps as their main source of water when accessible because other sources are no longer available. These findings are important as the National Water Policy of 2002 promotes the use of improved water sources, including the public taps, through user pay systems without adequate consideration of the impact of these costs on compliance. Through KIIs and FGDs it was clear that water shortage was a barrier to handwashing with soap and water, explaining that it is a difficult and expensive practice to maintain when water is scarce and or must be purchased.

Incorporation of participatory methods and ensuring community participation right from the inception stage of the project were significant contributing factors to obtaining improved understanding of community perceptions and their decision-making processes. The KIIs and FGDs conducted to triangulate the data obtained by questionnaire survey provided an insight into the understanding of the community regarding key issues relating to the availability, suitability, and appropriate use of the water services available. The qualitative findings highlighted potential entry points for effective control of childhood gastrointestinal infections. The use of participatory methods and community engagement provided an insight into community perceptions regarding unhygienic practices and the effective use of available resources. Compiling and analyzing community perceptions is the key determinant for successful adoption of co-designed interventions.

### Timor-Leste: Participatory Approaches to Learning About Animal Disease, Household Food Choices and Food Safety

Timor-Leste is a young, post-conflict country in Southeast Asia with a population of 1.27 million people in 2018 (79). Infrastructure is still rudimentary in many rural areas, and development is hindered by the challenging terrain and climatic conditions in much of the country (80, 81). Timor-Leste suffers high rates of child undernutrition, with 46% of children under five suffering from stunting in 2016, and children have low consumption of nutrient-rich animal-source food (82). In rural regions of Timor-Leste, heavy reliance on subsistence agriculture creates strong seasonal patterns in food availability and consumption (81). Household food insecurity exists when crop stores have been exhausted, and growing crops are not ready to be harvested (36). These patterns of crop availability, as well as seasonal foraging for wild vegetation, have been documented in parts of Timor-Leste (81, 83), however little data existed regarding the seasonality of animal-source food consumption, or consumption of non-domesticated animal species.

A research project on the impact of improving village chicken production on human diets and nutrition was carried out between April 2015 and June 2017 in response to the low frequency of consumption of animal-source food (84) and high levels of indigenous chicken ownership in Timor-Leste (85). This research project employed mixed methods through a gender-sensitive lens to collect qualitative data from three rural villages in the eastern, central, and western regions of the country. These villages were involved in a pilot ND vaccination program for village chickens between November 2014 and January 2017, with quantitative data being collected from the three pilot villages and three matched control village not vaccinating against ND. The research project was conducted through The University of Sydney in collaboration with the Timor-Leste Ministries of Agriculture and Fisheries and Health and funded through The University of Sydney and the Australian Government, while the ND vaccination program was a collaboration between the Australian Department of Foreign Affairs and Trade and the Timor-Leste Ministry of Agriculture and Fisheries, and was funded by the Australian Government.

Participating households (56–71 households per village) were selected on the basis of having one child under the age of 2 years at the time of enrolment and were followed longitudinally for just over 2 years. Quantitative data on chicken flock management were collected monthly in pilot villages, as well as dietary diversity data and anthropometric measurements from mothers and children seasonally from all six villages. This study involved participation by consultation, where the external researcher identified problems and potential solutions though gathering qualitative data on household food availability, infant, and young child (IYC) feeding and chicken flock response to ND vaccination through annual KIIs and FGDs. Key informants included village and sub-village heads, cultural leaders, local and municipal health, and agricultural staff. Focus group discussions involved both young and old members of the community and were sex-disaggregated.

While Timor-Leste is typically described as only having two seasons (a wet and a dry season), the seasonal calendars created through KIIs and FGDs identified three agriculturally important seasons: the dry season; wet season; and less-wet season. This finding informed the timing of collection of quantitative data for this study and allowed a more nuanced study of seasonal impact on diets and animal-source food (ASF) consumption. Through the quantitative study, adult dietary diversity was found to be significantly lower in the dry and wet seasons compared to the less-wet season. The qualitative study complemented the quantitative study by exploring the reasons behind the differences in food consumption through the year, and revealed both seasonal and non-seasonal drivers for household animal-source food consumption (7). In these rural areas of Timor-Leste, most animal-source food consumption was reported to occur during social events. Non-seasonal events included marriages, illnesses or deaths, and events occurring at fixed times, such as national holidays. Seasonal events included the consumption of ASF when guests visit, typically during the dry season, and ritualistic offerings for maize planting and harvest occurring at the start and finish of the rainy season. Local chicken is the most frequently consumed animal-source food, due to their significance in sociocultural practices, as well as their availability and the preference for the taste and texture of local chicken meat. Other animal-source food consumption practices also follow a seasonal pattern due to changing environmental conditions. Some farmers consume more eggs during the dry season, when decreased foliage increases chick predation. Where allowed, the hunting of non-domesticated animals was found to be a common practice amongst men and boys, and occurred more frequently during the dry season.

Triangulation between qualitative and quantitative findings identified an animal-source food consumption practice that has public health implications due to food safety. Livestock are valuable household assets: in many LMICs, the slaughter and consumption of livestock during disease epidemics is a common way for households to mitigate losses. In this study, quantitative data showed that household chicken consumption increased by 10–35% (*n* = 30–77) during ND outbreaks, and KIIs and FGDs confirmed that slaughter and consumption of sick birds, or consumption of recently dead birds in good condition were common practices. For zoonotic poultry diseases that result in higher morbidity and mortality rates in humans, such as HPAI, this practice could pose a significant public health risk. Programs that reduce the prevalence of fatal endemic diseases of poultry and livestock not only increase the numbers of healthy animals, but may also reduce consumption of sick animals and zoonotic disease transmission.

Quantitative analysis of IYC diets and qualitative exploration of IYC animal-source food feeding practices revealed that although eggs were considered culturally acceptable foods for IYC and parents preferentially gave eggs to IYC over adults, meat was considered texturally too tough for IYC to digest. This has implications for livestock interventions aiming to increase the availability of meat for household consumption, particularly if the improvement of child nutrition is an intervention target.

Finally, over the course of this 2-year longitudinal study, the researchers observed changes in the enthusiasm of female participants to engage during FGDs. In contrast to male participants, women were initially reluctant to voice their opinions or concerns. Repeated visits over a longer timeframe fostered familiarity and trust between the researchers and the participants, and women were able to speak more freely at subsequent FGDs and so achieved interactive participation (86).

Key findings from this study were reported back to stakeholders, with separate meetings conducted with the Australian Government, the Timor-Leste Ministries of Agriculture and Fisheries and Health at national and regional levels, and with village leaders and participants. Findings were also presented to a wider group of stakeholders in Dili, including researchers, multilateral organizations, and local and international NGOs.

### Uganda: Adapting PE to Understand Human Malnutrition

The Republic of Uganda is a landlocked country in East-Central Africa (87). It is bordered to the east by Kenya, to the north by South Sudan, to the west by the Democratic Republic of the Congo, to the south-west by Rwanda, and to the south by Tanzania. The southern part of the country includes a substantial portion of Lake Victoria, shared with Kenya and Tanzania. Uganda is in the African Great Lakes region. Uganda also lies within the Nile basin, and has a varied but generally a modified equatorial climate. In 2013, over a third of young children were stunted, 6% wasted, 14% underweight, 49% anemic, and 38% were deficient in Vitamin A (88).

As presented above, the early development of PE occurred largely in remote pastoralist areas of east Africa, and a recent adaptation of experiences among veterinarians in the 1990s and 2000s was to use PE methods to improve understanding of acute malnutrition in children and mothers in Karamoja, Uganda (89). In common with many other pastoralist areas, Karamoja has long been characterized by unacceptably high levels of acute malnutrition in children, despite significant investment in human nutrition and food security programs in these areas over many years. In 2016, there were 24 “information-giving” nutrition projects or programs in Karamoja implemented by 17 organizations, but the level of global acute malnutrition was increasing (90). An initial, informal review of programming approaches and types of nutrition intervention in Karamoja indicated three possible weaknesses. First, implementing agencies seemed not to consider the marked seasonality in livelihoods and food availability in Karamoja; conventional nutrition surveys were conducted twice a year and provided point prevalence estimates for global acute malnutrition (GAM), but provided limited information on monthly or seasonal variation in GAM. Second, Karamoja was experiencing important changes in livelihoods, with many households with low livestock ownership. Third, the knowledge and experience of women in project design had been overlooked, and there was limited understanding of women's perceptions of the main causes of acute malnutrition or their preferences for nutrition interventions.

With this context in mind, in 2018 an analysis employing consultative participatory tools was designed that aimed to describe the seasonality of acute malnutrition in Karamoja, and women's knowledge on the cause of acute malnutrition. The study was funded by USAID, UK Aid, and Irish Aid, implemented in collaboration with the Karamoja Resilience Support Unit and had two main phases. There was an initial ethnographic phase to document how women in Karamoja described malnutrition and related factors in their own language. Then, drawing on the initial phase, two PE methods were designed. First, a monthly calendar method enabled women to illustrate monthly variations in rainfall, availability of main food types, workload, human births, human diseases, and acute malnutrition. This method was designed to compare monthly changes in these variables, and women were provided with 100 counters for each variable and asked to distribute the counters by month. Therefore, the method showed monthly patterns of each variable using a standard, but arbitrary scale, and did not aim to produce absolute measures. Second, a causal diagram that involved scoring of the main causes of acute malnutrition and illustrating any important relationships between these causes.

**Figure 2:**
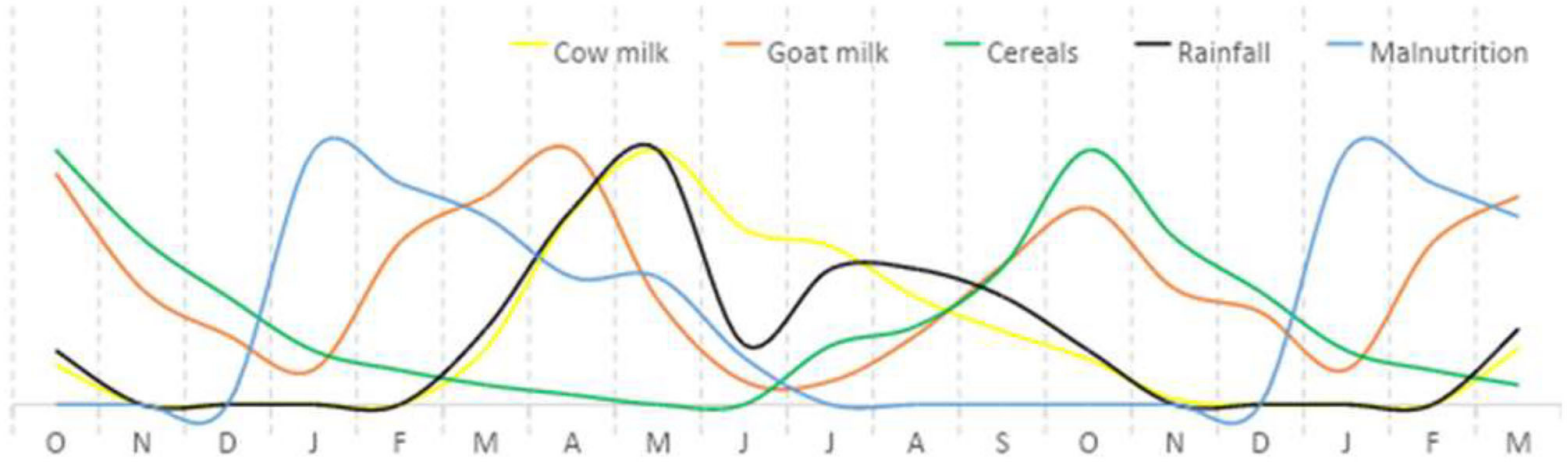
Example of a monthly calendar for a “typical year” for child malnutrition, rainfall, and availability of livestock milk and cereals for human consumption, Karamoja, Uganda, 2018. Monthly calendars with 16 women's groups; in each group each variable was illustrated by distributing 100 counters across 12 months; summated scores from all 16 groups were used to construct the diagram, and the y-axis scale is arbitrary; the monthly calendar method was based on the Gregorian, solar calendar with 12 months; this example was constructed over an 18-month period to enable comparison of trends over consecutive end of year periods.

**Figure 3:**
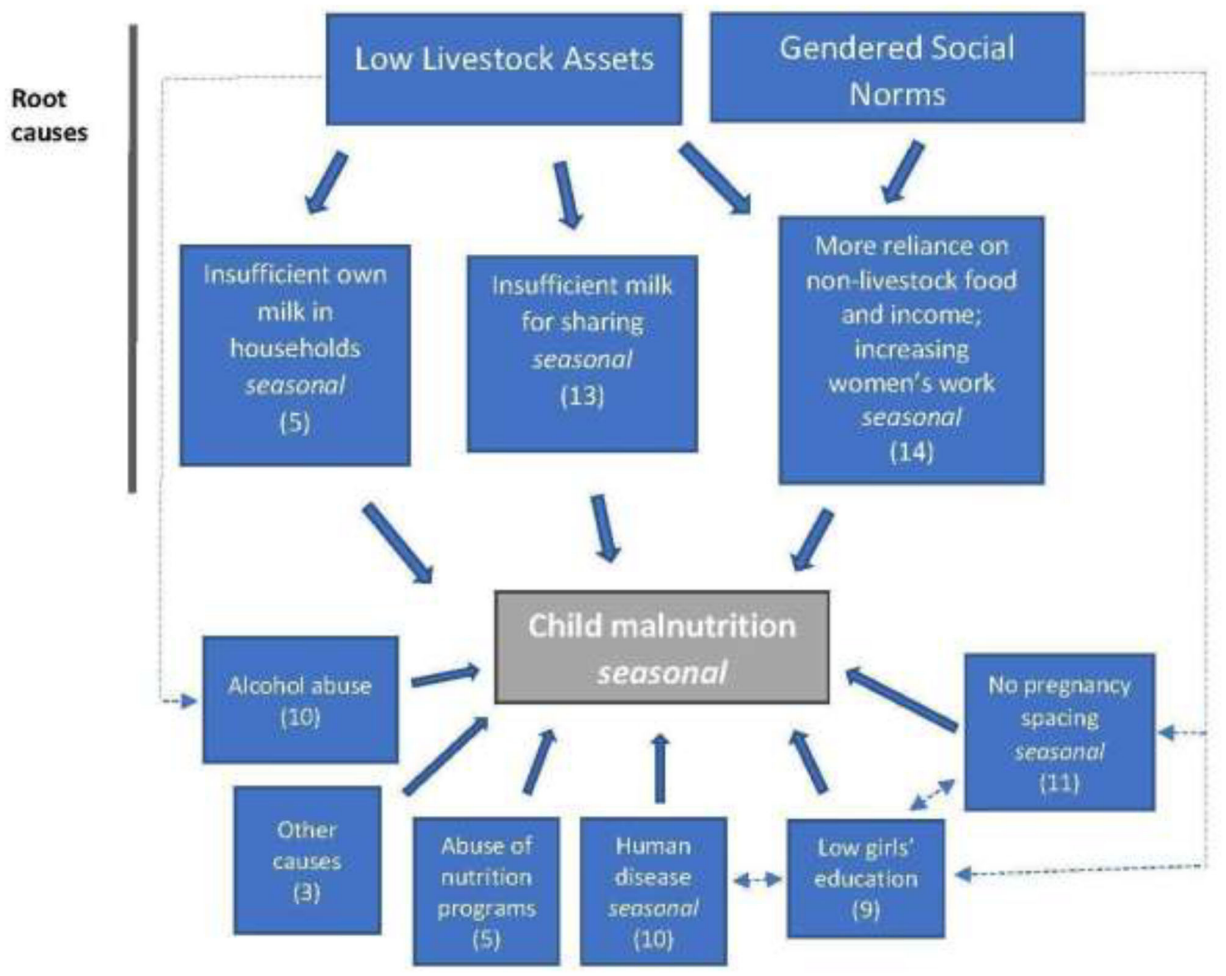
Example of a causal diagram for child malnutrition, Karamoja, Uganda, 2018. The numbers in the boxes are the median scores for the relative importance of the causal factors.

Among the key findings from this work was a hidden peak in acute malnutrition in January and February, which coincided with very limited availability of animal milk or availability of home-produced cereals. Nutritional status improved with the onset of rain, pasture growth, and resumption of milk production by livestock herds. This improved nutritional status was maintained and was supported by crop harvests toward the end of rainy months (Figure 2). As nutritional surveys were usually conducted in November or December, and then June or July, the surveys did not capture the peak in acute malnutrition in January and February. Women provided credible accounts of the causes of malnutrition (Figure 3). They explained that they thought malnutrition had two root causes: (i) the limited availability of livestock and milk; and (ii) social norms that overburden women with childcare responsibilities and finding and preparing food for the family. The women felt that these two root causes were interlinked and led to other issues and problems. Significantly, limited livestock ownership had a direct impact on food availability due to insufficient milk supply, but in a social context in which women were responsible for feeding the family. As households were forced to find more non-livestock sources of food and income, most of this burden fell to women. The non-livestock activities included crop production (frequently on small plots and with a high risk of rain failure) and a range of other activities that involved substantial effort for meager reward, and which hampered childcare. While women worked, their unweaned children remained at home under the care of siblings or other household members, with inadequate or no milk available to nourish them. Additional issues were linked to livestock-gender root causes, for example the loss of cattle affected men by negatively impacting on their self-identity and sense of purpose, and enabled them to spend more time in villages than previously with increased consumption of local brew and hard liquor. From the women's perspectives, this increased the risk of violence against them, and the likelihood unplanned pregnancies.

This example of PE methods showed how PE could be used to describe and explain multiple and complex food production and social factors that cause acute malnutrition, and which were difficult to capture using conventional nutritional surveys.

### Vietnam: Application of PE to Avian Influenza and Antimicrobial Resistance Control

Vietnam in southeast Asia is a mountainous country bounded by China to the North and Laos and Cambodia to the West (91). With a population of more than 95.5 million, Vietnam has seen rapid economic growth since major economic and political reforms in 1986, transitioning it from a low income to a rising lower middle-income economy. The poverty rate is now below 6% (92). An agricultural policy of decollectivization commencing in 1988 allowed rural households to take long-term contracts on land, and rent or buy capital stock and working capital. This policy shift away from cooperatives has been credited for the return to family farming in Vietnam (93). Nearly 40% of land in Vietnam is dedicated to agricultural production and 43% of the population are engaged in agricultural activities (94).

Livestock-keeping in Vietnam is characterized by smallholdings; 89% of farms are small family farms and on average, pastoralists own 1.7 tropical livestock units (94). Livestock are often secondary sources of income after rice and other crops but nonetheless, form an important part of agricultural livelihoods. Overall, livestock account for around 5.9% of Vietnam's gross domestic product (95). According to the World Organization for Animal Health (OIE) Performance of Veterinary Services (PVS) report (96) the veterinary services in Vietnam continue to face many and complex challenges spanning governance (chain of command), training of veterinarians and paraveterinarians, and physical, financial and human resources. The OIE PVS report, itself developed in a participatory manner with the veterinary services, describes the need for improvements in stakeholder engagement as one of three cross-cutting priorities for improvement of the veterinary service. Participatory epidemiology offers a low-input approach to putting primary stakeholders, livestock-keepers at the center of animal disease research and development (2). This section outlines how PE was used to tackle the challenges of HPAI, and more recently, antimicrobial resistance (AMR) in Vietnam, and describes how PE displayed particular utility in better understanding formal and informal communication channels between farmers and other animal health stakeholders, enabling effective design of ensuing research and interventions.

In 2003, Vietnam saw the beginning of a devastating HPAI epidemic. In a country dominated by smallholder poultry systems, surveillance was a daunting task. In order to understand the ways information about suspect HPAI cases flowed, between 2012 and 2013, CIRAD-The French Agricultural Research Center for International Development, the Hanoi University of Agriculture, the Nong Lam University and the National Institute of Veterinary Research of Vietnam engaged multiple stakeholder groups in a consultative PE process. Focus Group Discussions including semi-structured group interview and PP with poultry farmers were used as a starting point. Further participants were identified by snowball; farmers were asked who they communicated with when they suspected HPAI and when they mentioned a new participant group, the research team asked for any particular names. The team then contacted these people for interview. Proportional piling was used to quantify the relative likelihood of sharing information with each participant group mentioned. Groups identified included both people in public roles (government veterinarians) and private roles (feed and chick sellers, veterinary medicine sellers, veterinary technicians of feed companies, and pharmaceutical companies). Importantly, it was found that people in private roles had greater access to information in the face of suspect HPAI outbreaks compared with the government surveillance system, which “appeared as peripheral in the information sharing network” despite mandatory reporting. In fact, the local private workers were largely responsible for spreading the information to distant areas, acting as somewhat of an early warning system to farmers. Using this snowball technique to follow the flow of information, it became apparent that to enhance passive surveillance of HPAI there was a need for greater communication links between private and public veterinary services (97). Building on this study, further PE approaches were used to document the perceived benefits and costs of a passive surveillance system for HPAI. The authors explained, PE was useful for integrating economic and non-economic costs and benefits as well as stakeholder perceptions. Farmers were found to face uncertainty in transaction and outcome costs associated with notifying the government of suspicious cases. In this PE process, while the researchers defined the research problem, the truly consultative nature of their approach was evidenced by how they listened to stakeholders, basing their recommendations on the stakeholders' responses. A key recommendation to the government was consistency in response to notification, such as rules for compensation. One of the benefits of engaging multiple stakeholders in this approach was that it highlighted some agreement in perceived costs to reporting; all stakeholders (farmers, veterinary authorities, and private, upstream participant groups) anticipated a drop in market prices if knowledge of HPAI suspicions were released. The findings suggested that the benefits for all stakeholders to report disease outweighed the benefits of silence only if the market for selling diseased animals did not exist. The recommendation arising from this finding was that the poultry value chain needed greater quality control (98). The case of HPAI in Vietnam demonstrates the harmony between PE's “ground up” approach and the interrogation and augmentation of passive surveillance. As in South Sudan, PE enhanced the under-resourced government surveillance system.

In recent years, many stakeholders involved in the HPAI response in Vietnam have been involved in responding to other One Health challenges (99) including important emerging infectious diseases caused by antibiotic-resistant bacteria. Antimicrobial resistance, the ability of microbes to evade antimicrobials and therefore render them ineffective, is a natural phenomenon but is rapidly increasing due to the overuse and misuse of antimicrobials in humans and animals. Veterinarians, animal caretakers, doctors, and their patients are called to be better stewards of antimicrobials, to slow the increase in AMR. Described as one of the greatest health threats of our time (100), AMR is considered a priority challenge for human and animal health sectors in Vietnam (101). Overuse and misuse of antimicrobials does not occur in a vacuum. Especially in LMICs such as Vietnam where antimicrobials can often be purchased with no prescription, an understanding of the social and socio-economic context is crucial to designing better policy and implementing change (102). To this end, two initiatives, one in Southern Vietnam, and one in Northern Vietnam have applied participatory approaches and methods to the challenge of veterinary AMR. Both approaches engaged multiple stakeholders and explored antimicrobial use from economic and non-economic angles.

In Northern Vietnam, from 2016 to 2018, PE methods were used in a sequential mixed methods design to understand and look for ways to improve veterinary antimicrobial stewardship in family farming. The study was led by The University of Queensland and the International Livestock Research Institute in collaboration with the Hanoi University of Public Health and Thai Nguyen University of Agriculture and Fisheries. The research was funded by the CGIAR Research Program on Agriculture for Nutrition and Health and The Australian Government's Research Training Program. As in the HPAI study above, farmer FGDs were used as a first step, to identify further relevant participant groups and identify themes for further study. Farmers were asked a broad question, “What happens when a pig gets sick on your farm?” As the farmers mentioned the steps taken by themselves and other people they interacted with, they were written on cards to prompt discussion. In contrast to the study in southern Vietnam below, consensus was not sought. Rather, the group was probed to elicit the greatest diversity of responses to sick pigs in different scenarios until no new responses were provided (saturation). The reasons for various steps taken, for example, financial or physical constraints, beliefs and experience, were taken forward as themes for further study. Participant groups identified in the FGDs were also interviewed using semi-structured interview. The main groups identified were government veterinarians and private community animal healthcare workers. The findings were used to develop semi-quantitative survey tools for farmers and the additional participant groups identified. After the implementation of the survey and preliminary data analysis, the findings were brought back to the community for interpretation and development of a list of proposed interventions. This final stage included PE activities with individual participant groups, followed by a combined workshop of farmers, private, and public animal healthcare workers. Through this process, points of convergence and divergence were explored, and an agreed list of proposed interventions to improve antimicrobial stewardship finalized. As in the HPAI study, there were many points of common understanding and agreement. The community then presented these agreed recommendations to local and regional government and other external stakeholders. Using this adaptive, multi-stage process, the engagement of community groups moved from consultative participation in the first FGDs to participation in information giving in the surveys, and finally toward interactive and functional participation in joint analysis and proposal of interventions to external stakeholders including local authorities. The process was still dependent on external facilitators. Major decisions regarding governance of antimicrobials were proposed to be made by those people in positions of power, external to the community. However, some local decisions and plans, such as those to improve animal husbandry, were made by the participants. Through maintaining farmers as central stakeholders in the research and including other groups the farmers identified as important, this participatory process highlighted opportunities to improve antimicrobial stewardship that were agreeable to all (103).

More recently, from December 2017 to March 2018, during a Wellcome Trust-funded study in the southern Mekong Delta region of Vietnam, poultry farmers, veterinary drug shop owners, government veterinarians, and animal healthcare workers were engaged in a two-stage, mixed consultative PE and Q-sorting process. The first stage was “collective interview,” considered by the authors as a more appropriate term than focus group because the groups were heterogenous and they were seeking consensus rather than exploring alternative points of view. Before consensus was sought, however, PE methods were employed to allow participants to freely explore topics. Interview guides were semi-structured and PE methods included pairwise ranking, timelines, PP, and flow-charts to characterize poultry diseases, their prevention and control, identify sources of advice, and determine the timing and positive and negative opinions around antimicrobial use. These themes were chosen by the researchers based on knowledge of antimicrobial use and AMR. These data were used to develop a series of statements for use with a Q-sorting tool. This tool involved individual participants indicating on a scale how strongly they agreed or disagreed with each statement. The interviews allowed a more nuanced understanding of diversity of opinions and also provided opportunities for triangulation to verify findings, an important aspect of PE (104).

## Discussion

Within the veterinary arena, participatory epidemiology emerged in the final two decades of the twentieth Century, initially in association with small-scale, community-based development projects and subsequently playing a key role in the global effort to eradicate rinderpest (4). Working with communities in areas where animal health services were weak or non-existent was vital to understanding disease dynamics and opportunities to implement cost-efficient control programs (1). Over two decades later, resource shortages and competing priorities mean that vaccine-preventable animal diseases continue to kill huge—and in many cases undocumented—numbers of animals across the globe (105, 106), antimicrobial resistance has grown significantly (107) and food insecurity is rising (108).

Participatory epidemiology capitalizes on what is known and encourages communities to use their own knowledge of and skills with the animals they keep, the infectious diseases affecting their animals and the human diseases which can be acquired from their animals and vice versa. Indigenous knowledge which emerges from the experience of keeping the animals over long time periods enables animal keepers to define the clinical signs, salient lesions and epidemiological behavior in their own words which frequently have parallel meaning with technically employed terms (109). A failure to incorporate this local knowledge and experience may result in wrong conclusions and interventions which can fail to effectively and sustainably address the problem. Therefore, participatory epidemiological research provides a more comprehensive and diverse knowledge relevant for catalyzing positive change in the community toward solving their own problem in sustainable manner (25). The type of the approach and methods used in participatory epidemiology, when correctly employed, ensure inclusion in terms of gender, and different levels of education of the participants in seeking solutions to community problems. This enriches the information gathered (including more convention epidemiological data) and makes it specific to that locality, which are both important aspects in designing appropriate problem-solving strategies.

Participatory approaches provide an opportunity for all involved to agree on objectives. For example, in both Pakistan and Indonesia, endemic diseases such as haemorrhagic septicaemic and rabies were key priorities for communities, while in Timor-Leste, control of endemic ND may assist in decreasing the frequency of risky consumption practices. Openness by project funders and implementers to identify opportunities to control diseases of both local and global importance can help to build trust between participants and lead to more sustainable disease prevention, surveillance and control. These findings are in line with recommendations by Allepuz et al. (3) that PE techniques be employed to enhance dialogue between producers and national veterinary services.

Participatory research for collaborative, just action is useful for establishing unity in research and development goals, indicators for monitoring, and for understanding why interventions may or may not have been successful, i.e., it can be integral to learning. The PE research on AMR conducted in Northern Vietnam is an example of where authorities were alerted to on-the-ground challenges and opportunities in controlling an important emerging infectious disease. Participatory methodologies are a crucial component of all research, interventions or programmes that are likely to be affected by multiple factors. The inclusion of gender-sensitive approaches during the application of PE techniques increases the likelihood that the perspectives of more marginalized households and more vulnerable household members are heard and acted upon (2, 23).

The effectiveness of the One Health approach to infectious disease control has been greatly enhanced by incorporating social scientists and relevant participatory activities involving multiple sectors as outlined in the case studies from Indonesia, South Sudan, Tanzania, and Vietnam. The US Centers for Disease Control and Prevention define One Health as a collaborative, multisectoral, and transdisciplinary approach—working at the local, regional, national, and global levels—with the goal of achieving optimal health outcomes recognizing the interconnection between people, animals, plants, and their shared environment (110). The recognition of the importance of the social elements in this definition has been a major advancement in One Health implementation. In addition to social and political scientists, economists can play a role in ensuring that key findings, relating to both technical and policy aspects, are effectively presented to and addressed by senior decision-makers as presented in the Indonesian case study.

The combination of growing pressures on planetary (111) and social (112) boundaries and desperately inadequate funding for agricultural (especially livestock and aquaculture) research and development (113)—now further impacted by the contraction of the global economy due to COVID-19—makes it even more important that all resources are utilized as efficiently as possible. Projects and programs that run over a longer timeline can learn from and respond to community knowledge and priorities and provide increased opportunities for findings to be incorporated into policy and policy implementation frameworks. Longer-running activities also have a greater chance of achieving truly interactive participation, leading to self-mobilization as envisaged by Pretty (16). The PE activities presented in the case studies from Indonesia, Pakistan, South Sudan, and Uganda largely employed participation by consultation. Engagement with specific communities was generally over a short period with the information extracted contributing to larger national goals but rarely discussed with participants subsequently. These PE activities tended to be associated with larger, time-bound projects designed without community engagement. In the Karamoja case study in Uganda, the general approach of human nutrition programs was top-down, with women not being consulted or listened to, but expected to adopt program messages and change their behaviors. The PE study was more participatory relative to other programs and the general development context, and the first time that women's views had been documented, which should be considered a positive step. By contrast, the PE activities presented in the Tanzanian, Timor-Leste, and Vietnam case studies ran over 2–5 years and involved ongoing engagement with the same communities. Information and analyses were presented back to communities for discussion and subsequent action. Where veterinary research initiatives are concerned, funds are rarely allocated to monitoring and evaluating collective action, so, even if some of the PE research leads to self-mobilization, it has rarely been measured and reported. Consequently, even with the best of intentions, PE research is often constrained to participation by consultation, due to external forces (e.g., funder priorities and timelines). In terms of supporting functional community mobilization, the findings from the case studies suggest that project longevity may be more important than the size of the budget. Shorter term, large budget projects, may generate information but such projects rarely lead to transformation at the community level. Designing projects that run over longer periods, incorporate collective and reflexive learning through continuous evaluation (114) and are adaptive in line with findings, are more likely to achieve functional participation that can lead to self-mobilization (115, 116).

The triangulation of information using a combination of synergistic qualitative and quantitative tools provides an excellent opportunity to assess the robustness of the data collected and frequently provides insights into why the findings arose. This was amply demonstrated in the case studies from Tanzania, Timor-Leste, and Uganda in relation to household nutrition security, a complex and challenging issue that is advanced through effective participation and collaboration between communities, government agencies, and research and development personnel. Similar approaches are being refined in relation to water, sanitation, and hygiene research and development activities (117). In relation to the use of PE in infectious disease and EID outbreaks, including laboratory diagnosis as a component of the suite of triangulation tools employed is important (36). This was amply demonstrated in the South Sudan case study where laboratory testing confirmed the presence of HPAI H5N1 and not Newcastle disease as had been suspected by local producers.

As summarized above, a significant number of educational and training materials on PE techniques are freely available online and mostly in English. The need for additional material has been recognized, as has increasing opportunities for discussions relating to the inclusion of PE activities into regular national veterinary services programs (3). The more robust use of gender-sensitive methodologies and a gender lens (such as routine gender-disaggregation of data collection and analysis, the application of same gender discussion groups, gender-sensitive training curricula and methodologies, and empowerment tools) would enable participatory approaches to better address issues of socio-economic-, gender- and language-based differences.

## Conclusions and Recommendations for the Way Forward

Development literature, and increasingly epidemiological literature, abounds with references to the importance and effectiveness of participatory approaches. Despite this, participatory approaches remain less commonly utilized and inadequately resourced compared to more top-down approaches which all too-frequently fail to be successful in the long-term. In many parts of the world, vaccine-preventable animal diseases remain uncontrolled, contributing to food and nutrition insecurity, foodborne disease and antimicrobial resistance. More determined adherence to the fundamental principles of participatory epidemiology that communities must be actively involved “in defining and prioritizing problems, and in the development of solutions to them,” as defined by Catley et al. (2), is vital. This will require adaptive management of projects that are of a sufficient duration for trust and effective collaboration to develop between partners. The incorporation of gender-sensitive participatory impact assessments into activities will assist with measuring the degree to which objectives have been met and key outcomes achieved for all stakeholders.

## Author Contributions

RA conceived the paper, took the lead with writing the general sections, and contributed to all sections. SA wrote the case study on Pakistan. AA wrote the study on South Sudan. BB contributed to the Tanzanian section study. TC wrote the section on Vietnam, AG and MH wrote the study on Indonesia. ER contributed to the Tanzanian study. JW wrote the Timor-Leste study. AC wrote the Ugandan study. All authors contributed to the revision process for all sections.

## Conflict of Interest

The authors declare that the research was conducted in the absence of any commercial or financial relationships that could be construed as a potential conflict of interest.
